# A tool for live-cell confocal imaging of temperature-dependent organelle dynamics

**DOI:** 10.1093/jmicro/dfad064

**Published:** 2024-01-12

**Authors:** Keiko Midorikawa, Yutaka Kodama

**Affiliations:** Center for Bioscience Research and Education, Utsunomiya University, 350 Mine, Utsunomiya, Tochigi 321-8505, Japan; Center for Bioscience Research and Education, Utsunomiya University, 350 Mine, Utsunomiya, Tochigi 321-8505, Japan

**Keywords:** confocal laser-scanning microscopy, microscope stage, organellar dynamics, organellar morphology, peroxisome, temperature control

## Abstract

Intracellular organelles alter their morphology in response to ambient conditions such as temperature to optimize physiological activities in cells. Observing organelle dynamics at various temperatures deepens our understanding of cellular responses to the environment. Confocal laser microscopy is a powerful tool for live-cell imaging of fluorescently labeled organelles. However, the large contact area between the specimen and the ambient air on the microscope stage makes it difficult to maintain accurate cellular temperatures. Here, we present a method for precisely controlling cellular temperatures using a custom-made adaptor that can be installed on a commercially available temperature-controlled microscope stage. Using this adaptor, we observed temperature-dependent organelle dynamics in living plant cells; morphological changes in chloroplasts and peroxisomes were temperature dependent. This newly developed adaptor can be easily placed on a temperature-controlled stage to capture intracellular responses to temperature at unprecedentedly high resolution.

Temperature influences various biochemical activities in living organisms, including plants [1,2]. For example, chloroplast function is temperature dependent: at low temperature and weak light irradiation, the lipid composition of chloroplast membranes changes, leading to reduced membrane fluidity, inhibited stomatal reactions and reduced activity of enzymes involved in a series of biochemical reactions [3]. Changes in membrane lipids also affect chloroplast morphology [4–7]. Indeed, in mutant plants with altered chloroplast membrane lipid composition, the chloroplast thylakoid membrane is highly curved and the envelope membrane is much rounder, resulting in balloon-like chloroplasts with large interstitial regions [4,6].

Peroxisomes are responsible for the photorespiratory metabolism associated with the photosynthesis of chloroplasts. Under weak light irradiation, peroxisomes are in close proximity to chloroplasts with morphological changes [8–10]. This suggests that organelle morphology and physical contact may alter metabolic processes in response to the external environment. Peroxisomes are highly dynamic organelles whose morphology and abundance immediately change in response to changes in the extracellular environment [11–15]. Therefore, investigating the details of the environmental responses of these dynamic organelles will increase our understanding of stress responses in plants.

Maintaining proper temperature control in specimens under the microscope is important when observing living cells and organelles [16]. The temperature of the specimen between the microscope glass slide and coverslip is greatly affected by ambient air temperature, direct contact with the objective lens via the immersion medium and the coverslip and heating due to illumination [17]. Confocal laser-scanning microscopy is an excellent tool for high-resolution imaging of organelles in live cells, but accurate temperature control is challenging. To control the temperature under the microscope, several custom-made microscopic stages have been developed [18,19]. Among these, Peltier-based temperature-controlled stages appear to accurately maintain specimen temperatures, but complete customization is not easy for many researchers. Peltier-based temperature-controlled stages are also commercially available, but they may not be sufficiently adiabatic to protect the specimen from the ambient environment [17].

Here, we report on a custom-made adaptor (Fig. 1a) that can be installed on a commercial temperature-controlled microscopic stage (Fig. 1b). Installation of this adaptor allowed us to precisely control specimen temperature under a confocal microscope. We demonstrated its effectiveness by detecting temperature-dependent morphological changes in chloroplasts and peroxisomes in the liverwort *Marchantia polymorpha*, whose organelles are clearly observable by fluorescence microscopy [20,21]. This adaptor offers a new tool for capturing temperature-related processes in various organelles of living cells at high resolution.

**Fig. 1.**
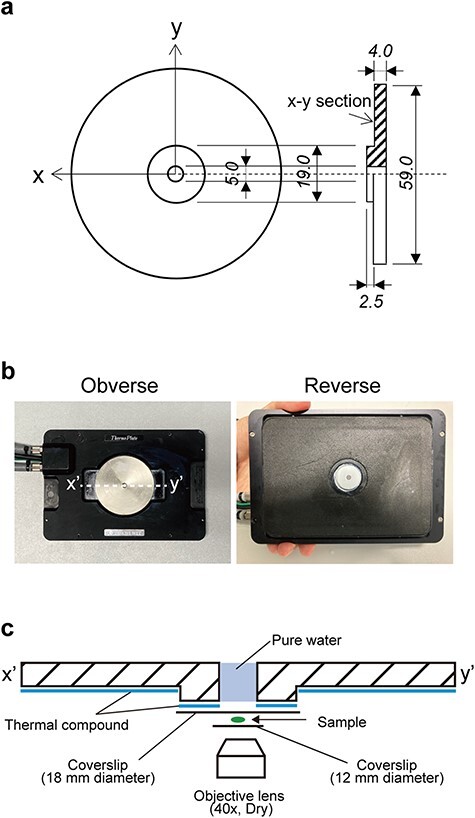
The custom-made adaptor for the temperature-controlled microscope stage. (a) Design of the adaptor. The adaptor is made of copper. Thermal compound is applied between the adaptor and stage to increase thermal conductivity. Dimensions are in millimeters. (b) Exterior of the adaptor. (c) Schematic diagram of sample (specimen) setting in the adaptor. The *x*–*y* cross section is shown in (b). Thermal compound is applied to the reverse side of the adaptor, and a round coverslip (18-mm diameter, 0.13- to 0.17-mm thick) is placed onto the compound. The specimen is placed in the center of the coverslip and covered with another round coverslip (12-mm diameter, 0.13- to 0.17-mm thick). The hole in the center is filled with pure water and observed using a ×40 dry lens.

Transgenic *M. polymorpha* expressing OEP7-Citrine [20] or Citrine-peroxisomal targeting signal 1 (PTS1) [21] were maintained asexually and cultured on half-strength Gamborg’s B5 medium with 1% (w/v) agar at 22°C under 75 μmol m^−2^ s^−1^ continuous white light. OEP7-Citrine consists of the N-terminal 50 amino acids of *Arabidopsis thaliana* outer envelope membrane protein 7 (AT3G52420) fused to yellow fluorescent protein (Citrine) [20], and Citrine-PTS1 is Citrine fused to PTS1 (Ser-Lys-Leu) at the C-terminus [21]. The light intensity was measured using a light meter (LI-250A; LI-COR Biosciences). One-day-old gemmalings (immature thalli grown from gemmae) obtained from ∼1 month-old transgenic thalli [21] were used for microscopy observation.

A confocal laser microscope (Leica TCS SP8X, Leica microsystems, Wetzlar, Germany) equipped with a hybrid detector and a flexible pulsed white light laser was used to observe Citrine and chlorophyll fluorescence. An HC PL APO ×40 dry lens was used as the objective lens to avoid contact with the coverslip. Citrine and chlorophyll were excited by a 513 nm laser. Emission signals were captured at 520–570 nm for Citrine and 650–750 nm for chlorophyll. When observing Citrine fluorescence, chlorophyll fluorescence was blocked using a time-gated imaging method (gating time set at 0.3–12.0 ns) [22].

For chilling treatment, the temperature was initially set at 22°C under light or dark conditions for 30 min and was changed to 5°C before starting fluorescent image acquisition. For light conditions, the specimens were kept under white light (50 μmol m^−2^ s^−1^) for 2 h by turning on the microscope light, normally used for observation with an eyepiece, during fluorescent image acquisition. Light intensity was measured with a light meter (LI-250A; LI-COR Biosciences, NE, USA). For dark conditions, specimens were kept for 2 h in the dark, except during fluorescent image acquisition. Images were captured every 10 min to generate a time series.

Images were processed using ImageJ software (https://imagej.net/ij/) [23]. Chloroplast circularity was measured manually on the basis of Citrine fluorescence at the chloroplast outer membrane (OEP7-Citrine line [20]). Peroxisome morphology was determined using the ‘Analyze Particles’ function of ImageJ. Images of peroxisomes labeled with Citrine (Citrine-PTS1 line [21]) were pretreated with a median filter to remove background irregularities by the Rolling Ball method prior to analysis [24]. Morphological analyses of both chloroplasts and peroxisomes were performed in triplicate independent experiments. All data were plotted using GraphPad Prism 10.0.3 (GraphPad Software Inc.), and statistical analysis was performed as described in the figure legends.

To accurately control cell temperature under a microscope, we developed an adaptor made of copper for installation on a commercial temperature-controlled stage (Thermo Plate^®^, TOKAI HIT, Shizuoka, Japan) (Fig. 1a and b). The specimen was placed on a large round coverslip on the central, raised region of the adaptor (Fig. 1c). The specimen was then covered with a smaller round coverslip, taking care not to shift the specimen from the center (Fig. 1c). The hole was filled with pure water to reduce the influence of ambient temperature (Fig. 1c).

To investigate the effect of the adaptor on controlling temperatures, we measured the temperatures in various points of the specimen holder (obverse or reverse side of the adaptor and the microscope glass slide) using infrared thermography (InfReC Thermo FLEX F50B-STD, Avio, Yokohama, Japan) and confirmed that the temperature in the hole of the adaptor was the actual temperature (Fig. 2a and Supplementary Fig. S1). We examined the cooling rate of specimens on the microscope stage with and without the adaptor using infrared thermography. With the adaptor installed on the stage, the temperature dropped to almost the preset temperature in ∼10 min and stabilized in ∼30 min (Fig. 2b). When the temperature was measured using a glass slide without an adaptor at low temperature (preset temperature 5°C), the temperature on the glass slide only dropped to 9°C even after 40 min, deviating by ∼5°C from the preset temperature (Fig. 2b and Supplementary Fig. S1). We then examined the heating rate of the stage with and without the adaptor installed. At the high preset temperature of 37°C, the temperature of the stage with the adaptor installed reached almost the preset temperature in 20 min, while the temperature of the stage without the adaptor rose to 37°C, but it took 40 min to reach it (Fig. 2b and Supplementary Fig. S1). A comparison of temperature fluctuations with and without the adaptor during a 10-min period starting 30 min after setting the temperature showed that the coefficient of variation was 0.47% with the adaptor and 0.72% without the adaptor at a preset temperature of 37°C. At a set temperature of 5°C, the difference was even larger: 2.91% with and 7.41% without the adaptor (Table 1). These results indicate that the adaptor improves both the cooling and heating rates, enabling more accurate temperature control in cells, especially at low temperatures. When the temperature was set to 37°C or 5°C with the adaptor attached, the temperature was maintained even after 360 min, highlighting the strong temperature stability of the stage with the adaptor installed (Fig. 2c).

**Fig. 2.**
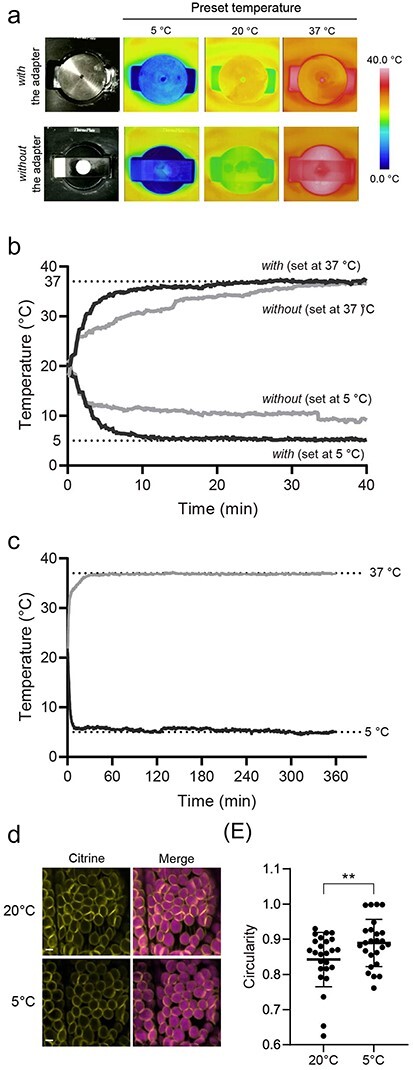
Improvement of the cooling and heating rates using the microscope stage adaptor. (a) Temperature measurement by infrared thermography. The upper images show the stage with the adaptor, and the lower images show the stage with only the glass slide without the adaptor. (b) Temperature of the stage during the 40 min after changing the temperature setting of the microscope stage from 20°C to 37°C or 5°C. ‘with’ and ‘without’ indicate the stage temperature with and without the adaptor, respectively. (c) Changes in temperature over 360 min on the stage with the adaptor installed after changing the temperature setting from 20°C to 37°C or 5°C. (d) Visualization of morphological changes in chloroplasts under low-temperature conditions using transgenic *M. polymorpha* cells expressing OEP7-Citrine. One-day-old gemmalings were incubated for 90 min at 20°C or 5°C on the stage with the adaptor installed. Bars, 3 µm. (e) Quantification of chloroplast morphology at 20°C and 5°C. Data are means ± SD (*n* = 25); significant differences were determined using the paired *t*-test (two-tailed). **P < 0.01.

**Table 1.** Temperature fluctuations of the microscope stage during a 10-min period starting 30 min after setting the temperature

Adaptor	Preset temperature	Mean	Standard deviation	Coefficient of variation
*with*	5°C	5.20	0.15	2.92%
*without*	5°C	9.67	0.72	7.41%
*with*	37°C	37.01	0.17	0.47%
*without*	37°C	36.37	0.26	0.72%

Previous studies have shown that the chloroplast outer envelope assumes a nearly spherical morphology at low temperatures [7,20]. To confirm the effectiveness of the stage with the adaptor installed, we analyzed changes in chloroplast morphology in *M. polymorpha* in response to low temperatures. For this analysis, we used transgenic *M. polymorpha* cells expressing OEP7-Citrine fusion protein, which localizes to the chloroplast outer envelope membrane [20,25,26]. When transgenic cells were incubated on the stage with the adaptor installed in the dark for 90 min at either 20°C or 5°C, chloroplast sphericity was higher at 5°C, confirming that changes in chloroplast morphology at low temperature can be induced in living cells on the stage with the adaptor (Fig. 2d and e).

Chloroplasts share several metabolic pathways with peroxisomes, suggesting that the environmental responses of these two organelles are closely related [27,28]. Since temperature-dependent morphological changes were observed in chloroplasts (Fig. 2d and e), we examined whether changes in morphology could also be observed in peroxisomes using transgenic *M. polymorpha* cells expressing Citrine-PTS1, which localizes to peroxisomes [29,30]. Again, we used the stage with the adaptor installed. Because light-dependent morphological changes in peroxisomes have been reported [8], we incubated 1-day-old gemmalings on the microscope stage under light or dark conditions with chilling at 5°C for 90 min and observed the fluorescent peroxisomes after incubation (Fig. 3a). We compared the aspect ratio under each condition as a parameter for morphological evaluation. The aspect ratio was significantly higher in the chilling/light treatment group than in the chilling/dark treatment group (Fig. 3b). Comparing the cumulative frequency distribution per cell, in chilling/dark-treated cells, only ∼10% of all peroxisomes had an aspect ratio of 2.0 or higher, whereas in chilling/light-treated cells, ∼30% of all peroxisomes had an aspect ratio of 2.0 or higher (Fig. 3c).

**Fig. 3.**
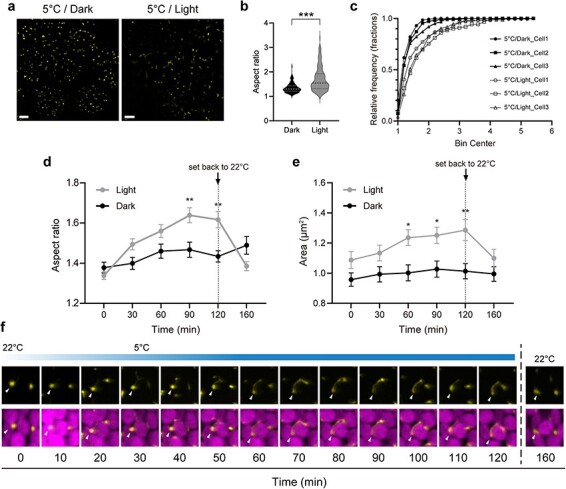
Observation of peroxisome dynamics under chilling stress using the microscope stage adaptor. (a) Visualization of peroxisomal morphology in transgenic *M. polymorpha* expressing Citrine-PTS incubated under dark (left) or light (right) conditions at 5°C for 90 min. The fluorescent signals indicate Citrine-labeled peroxisomes. Bars, 10 µm. (b) Distribution of the aspect ratio of peroxisomes. Medians are shown by dashed lines, and 25th and 75th percentiles are indicated by the lower and upper dotted lines, respectively [*n* = 128 (dark) and *n* = 180 (light)]. Statistical significance was determined using the Mann–Whitney *U*-test. ***P < 0.001. (c) Peroxisomal aspect ratio in cells exposed to dark and light conditions shown as cumulative frequency distributions. Each line represents data from 100 peroxisomes sampled in each cell: *n* = 3 cells per group. (d and e) Changes in peroxisomal aspect ratio (d) and area (e) over time. Filled circles represent the mean ± SD of all peroxisomes in cells under each light condition. Specimens were placed on the microscope stage and acclimated to dark or light conditions at a stage temperature of 22°C for 30 min; the temperature was set to 5°C prior to observation. The temperature setting was maintained at 5°C for 120 min and then returned to 22°C. Observations after temperature return to 22°C were made 160 min after the start of measurements. Significant differences between light and dark conditions for each time point were determined using an unpaired *t*-test. *P < 0.05, **P < 0.01. (f) Visualization of changes in peroxisomal morphology over time. The gradient bar above indicates changes in stage temperature over time, as estimated from Fig. 2a. The upper panels show Citrine fluorescence only, while the lower panels show merged images of Citrine fluorescence with chlorophyll fluorescence. White arrowheads indicate specific peroxisomes demonstrating changes.

Analysis of a time series of morphological changes in peroxisomes showed that the aspect ratio and area of peroxisomes in the light-treated group gradually increased after exposure to low temperature (Fig. 3d and e and Supplementary Figs. S2 and S3). This morphological change in peroxisomes was not observed under chilling/dark conditions (Fig. 3d and e and Supplementary Figs. S2 and S3). After 90 min of treatment, both parameters were significantly higher in the light-treated group than in the dark-treated group (Fig. 3d and e and Supplementary Figs. S2 and S3). Detailed observation of individual peroxisomes showed that in chilling/light-treated cells, some peroxisomes that were labeled with Citrine began to protrude, and morphological changes were observed 20 min after setting the temperature (Fig. 3f). Several peroxisomes were observed to elongate along the chloroplasts after 30 to 120 min of treatment (Fig. 3f). This phenomenon was reversed as soon as the temperature was returned to 22°C (Fig. 3d–f), indicating that a combination of light and chilling induces morphological changes in peroxisomes.

Using our custom-made adaptor and strict temperature control, we observed morphological changes not only in chloroplasts but also in peroxisomes in response to light under chilling stress (Figs. 2d and e and 3a–3). While we utilized gemmaling cells of *M. polymorpha* in this study, the temperature control system should be applicable to various other plant species, tissues and cells. Our custom-made adaptor (Fig. 1a) is designed to be specifically installed on a commercial temperature-controlled microscope stage (Thermo Plate^®^ TP-CHSQ-C, TOKAI HIT, Shizuoka, Japan) (Fig. 1b). Currently, our custom-made adaptor cannot be installed on different types of commercial temperature-controlled stages. Our adaptor is a proof-of-concept tool: a similar adaptor must be specifically designed and produced for each type of commercial temperature-controlled stage. However, technically, customization of the adaptor would be easier than complete customization of the temperature-controlled stage. The shape of the adaptor would vary depending on the type of temperature-controlled stage, but the material should be copper. Copper is known for its high thermal conductivity and its cost-effectiveness.

Light-dependent morphological changes in peroxisomes have been reported previously and were shown to be dependent on photosynthesis [8]. In this study, we determined that a combination of light and chilling induces morphological changes in peroxisomes (Fig. 3a–f). Given that photosynthetic activity in chloroplasts is almost suppressed under low-temperature conditions in the presence of light irradiation [31], our results under light/chilling treatment are likely due to a different mechanism from photosynthesis-dependent morphological changes in peroxisomes. Peroxisomes are thought to approach chloroplasts in response to intense light stress and to scavenge excess reactive oxygen species originating from chloroplasts [32,33]. Even under normal conditions, peroxisomes are involved in photorespiratory metabolism associated with photosynthesis and interact closely with chloroplasts [8–10]. The morphological changes in peroxisomes observed in this study under chilling stress likely contribute in some way to a protective mechanism for photosynthetic function in plants exposed to light damage. The finding that morphological changes in peroxisomes are initiated within an hour after exposure to low temperatures (Fig. 3d–) suggests that these morphological changes in peroxisomes are one of the initial responses employed by plant cells to adapt to low-temperature environments.

Plants are thought to employ complex, diverse mechanisms to adapt to constantly changing environmental conditions in the field [34]. The difficulty of controlling the combined factors of light and temperature in a stable manner has led to various innovations in the equipment used for microscopy observation of plants in response to chilling stress [18,19]. In this study, as a proof-of-concept, we improved the temperature stability and precision of a commercial temperature-controlled microscope stage by combining it with our custom-made adaptor. We succeeded in capturing light- and temperature-dependent morphological changes in organelles in living plant cells. This adaptor is a powerful analytical tool for exploring temperature-dependent cellular responses in detail.

## Supplementary Material

dfad064_Supp

## Data Availability

The data that support the findings of this study are available from the corresponding author (kodama@cc.utsunomiya-u.ac.jp) upon reasonable request.
