# Supplementary material for: A tool for live-cell confocal imaging of temperature-dependent organelle dynamics
Source: Microscopy (Oxf). 2024 Jan 12;73(4):343–8. doi: 10.1093/jmicro/dfad064 (PMC11288189; doi:10.1093/jmicro/dfad064)
Supplement: dfad064_Supp [file dfad064_supp.zip › suppl_data/SuppIementary_Data_revised.docx]

**Supplementary data**

**A tool for live-cell confocal imaging of temperature-dependent organelle dynamics**

Keiko Midorikawa^1^, Yutaka Kodama^1^

^1^Center for Bioscience Research and Education, Utsunomiya University, 350 Mine, Utsunomiya, Tochigi 321-8505, Japan.

**Supplemental Data**

**
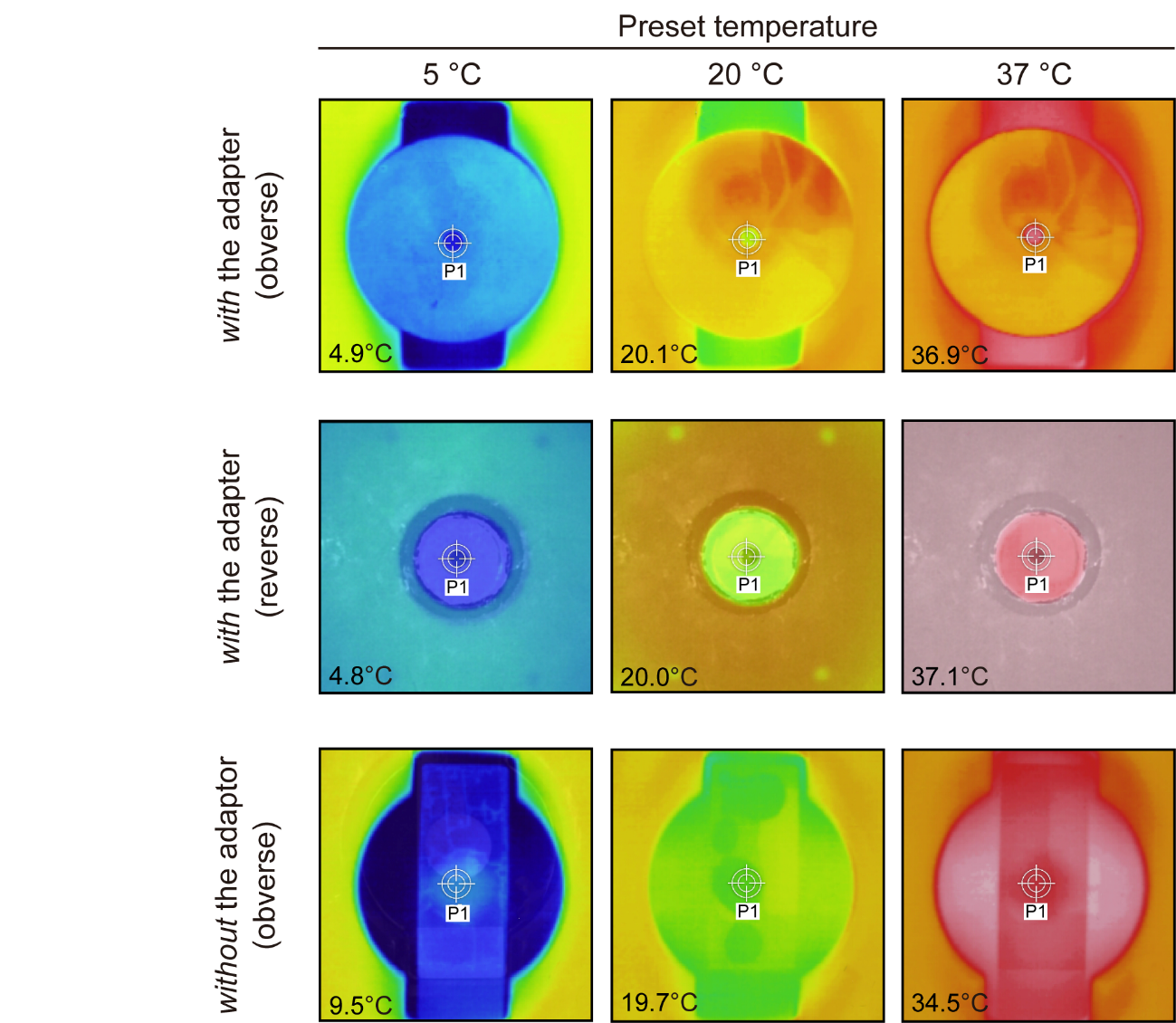
**

**Supplementary Figure 1.** Temperature measurement of the stage using infrared thermography. The top and middle images are thermographic images of the stage with the adaptor attached, and the bottom image shows the observation stage without the adaptor and with only a glass slide. The stage temperature was measured 40 min after the temperature setting was changed. The number in the lower left of each image indicates the temperature of the measurement point (P1) on the image.

**
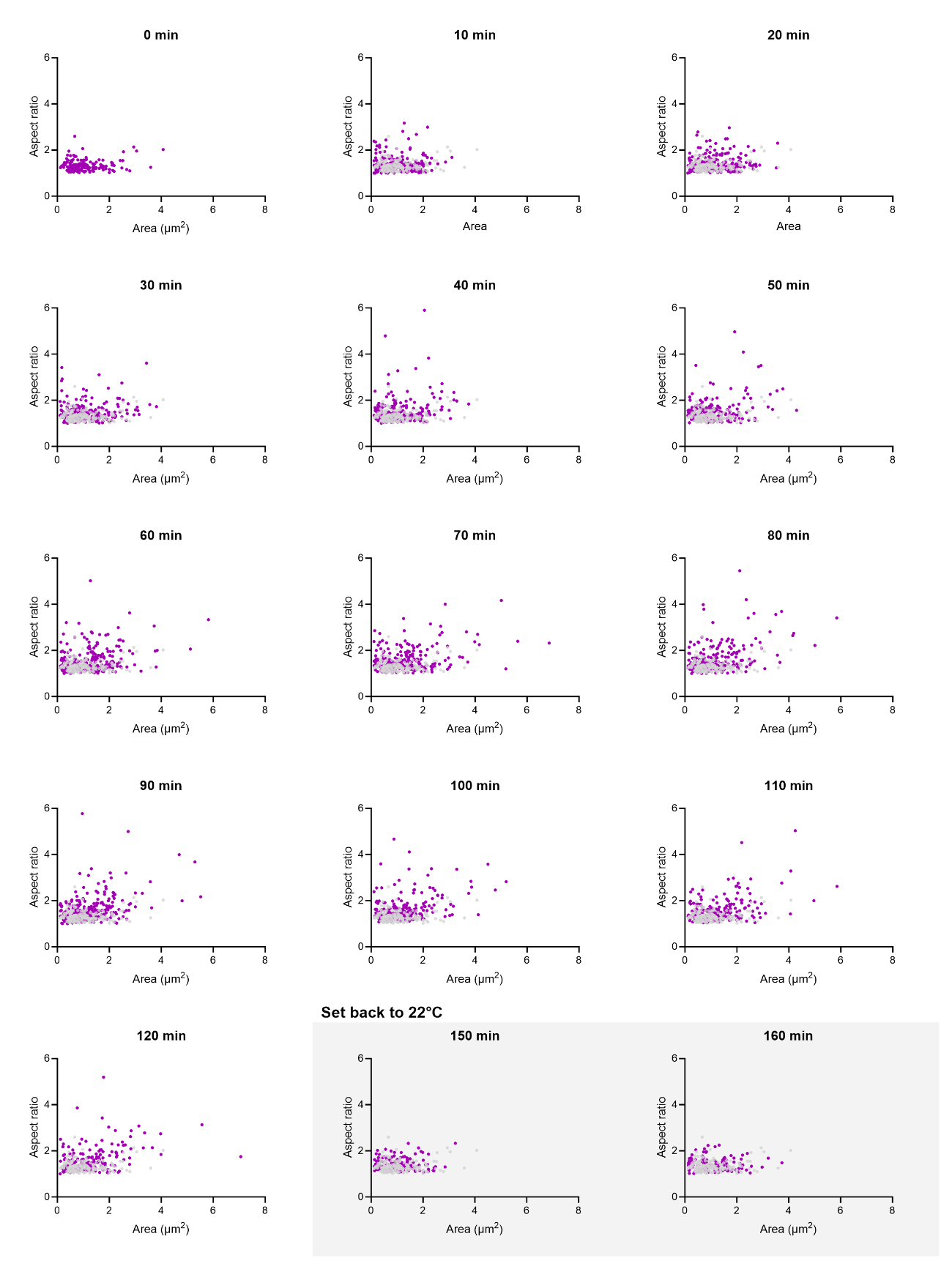
**

**Supplementary Figure 2.** Scatterplots showing morphological changes in peroxisomes over time in light-treated cells at 5°C. The vertical axis represents the aspect ratio, and the horizontal axis represents the area. To facilitate comparison with the initial morphology, a plot of peroxisomes at 0 min of observation is shown in gray and overlaid with the plots at other time points. Each dot represents an individual peroxisome.


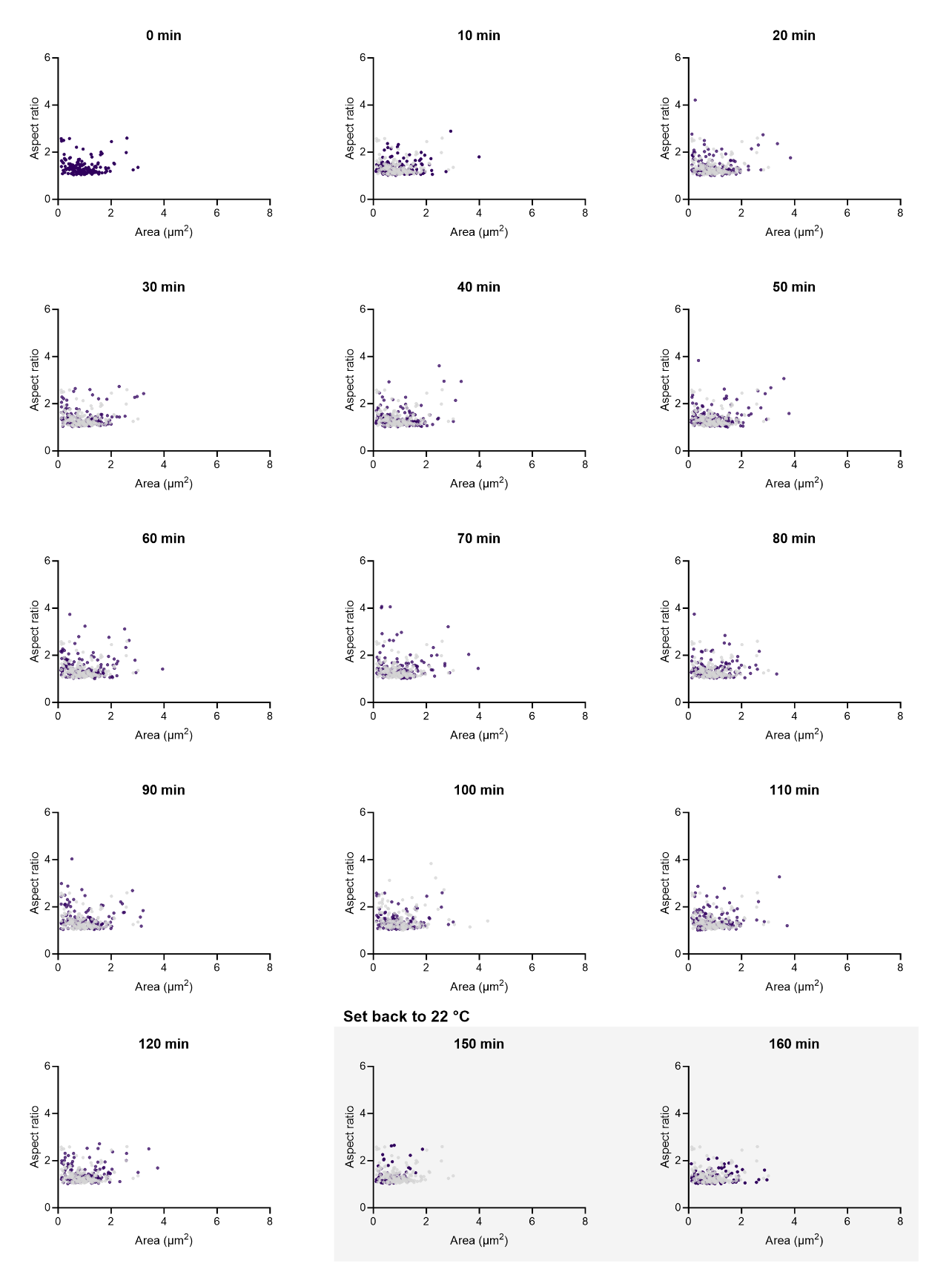


**Supplementary Figure 3.** Scatterplots showing morphological changes in peroxisomes over time in dark-treated cells at 5°C. The vertical axis represents the aspect ratio, and the horizontal axis represents the area. To facilitate comparison with the initial morphology, a plot of peroxisomes at 0 min of observation is shown in gray and overlaid with the plots at other time points. Each dot represents an individual peroxisome.
